# Observing Expertise-Related Actions Leads to Perfect Time Flow Estimations

**DOI:** 10.1371/journal.pone.0055294

**Published:** 2013-02-06

**Authors:** Yin-Hua Chen, Fabio Pizzolato, Paola Cesari

**Affiliations:** Department of Neurological, Neurophysiological, Morphological, and Movement Sciences, University of Verona, Verona, Italy; Duke University, United States of America

## Abstract

The estimation of the time of exposure of a picture portraying an action increases as a function of the amount of movement implied in the action represented. This effect suggests that the perceiver creates an internal embodiment of the action observed as if internally simulating the entire movement sequence. Little is known however about the timing accuracy of these internal action simulations, specifically whether they are affected by the level of familiarity and experience that the observer has of the action. In this study we asked professional pianists to reproduce different durations of exposure (shorter or longer than one second) of visual displays both specific (a hand in piano-playing action) and non-specific to their domain of expertise (a hand in finger-thumb opposition and scrambled-pixels) and compared their performance with non-pianists. Pianists outperformed non-pianists independently of the time of exposure of the stimuli; remarkably the group difference was particularly magnified by the pianists’ enhanced accuracy and stability only when observing the hand in the act of playing the piano. These results for the first time provide evidence that through musical training, pianists create a selective and self-determined dynamic internal representation of an observed movement that allows them to estimate precisely its temporal duration.

## Introduction

We are now witnessing a renewed interest in time perception, in particular in order to understand the processes that allow the evaluation of the passage of time given that perceived temporal duration is not isomorphic to physical duration and can be distorted by several factors [1]–[3]. There has been a growing interest in revealing how the information conveyed by a picture affects the perception of time [4]–[6]. For instance, when estimating the time of exposure of images portraying different dance body positions, individuals perceived a longer duration for those images that implied a greater amount of movement dynamics and vice versa. This suggests that an internal simulation of the implied action sequence influenced perceivers’ sense of temporal duration [5]–[6]. Interestingly, individuals showed higher accuracy in temporal duration discrimination for photographs representing athletes in active postures than the same athletes portrayed in a quiet standing position [4]. A growing body of evidence seems to sustain the notion that while temporal duration evaluation changes according to the dynamics of the action observed, having an internal embodiment of that action helps to estimate its temporal duration more accurately. However, little is known about the timing nature of these internal simulations and whether their accuracy changes as a function of the level of perceptual and motor skills acquired.

This tight link between temporal duration evaluation and internal action simulation has been shown to be critical in the understanding of other people’s behavior. Athletes represent a potentially enlightening example for their ability to anticipate the outcome of actions performed by other players [7]–[8]. Indeed, these motor experts are capable, through the “reading” of the kinematics of an action, to express a sophisticated internal neural mechanism to decode the action’s dynamical development [7]. Several studies already show that motor experts possess detailed internal action-specific simulations that permit fast and precise evaluations particularly for actions that pertain to their domain of expertise [7], [9]–[10]. In fact, this interpretation is based on the notion that the observation of an action, no matter if it is “implied” as in a static picture [11]–[15] or “apparent” as in a video-clip [16]–[19], induces muscle-specific brain activations as if the observed actions are internally performed. Moreover, this effect is even stronger when observers with motor-related experience view “familiar” than “unfamiliar” actions [17]–[19]. What is still not clear is whether these very effective internal simulations optimize their functioning when observers view the action presented in a picture rather than in a video-clip format. This would be of particular interest for a deeper understanding of the internal mechanism that is responsible for action imitation and learning [16]–[20].

To deal with these issues we asked professional pianists to reproduce different times of exposure of visual displays, which were specific/familiar (a hand in piano-playing action) and non-specific/unfamiliar (a hand in finger-thumb opposition) to their domain of expertise and compared their performance with non-pianists. Moreover, scrambled-pixels of the piano-playing action was served as a control stimulus since it is a neutral image without any action representation and it is equally (un)familiar to both pianists and non-pianists. The durations to be estimated were either longer or shorter than one second [21] and the visual displays were presented either in the format of picture or video-clip.

We hypothesized that just for pianists the observation of a piano-playing movement would evoke a highly specific internal action simulation [17], which would consequently increase the accuracy in reproducing temporal duration, compared to the observation of non-piano-playing movement. On the other hand, we expected no difference between pianists and controls for timing the non-piano-playing related stimuli, that is, finger-thumb opposition actions and scrambled-pixels. Furthermore, we had an open expectation of whether the internal action simulation would work differently if an action is presented in a picture format or in a video-clip format.

## Methods

### Participants

We recruited 15 professional pianists (7 males, mean age 23.7±4.3 years; with an accumulated lifetime practice experience of 17.2±4.6 years of daily practice) and 15 age-matched (24.3±4.0 years) musically naive individuals (6 males; with no formal instrumental training) as a control group. All participants were right-handed according to the diagnostic criteria of the Edinburgh Inventory [22]. They were naive to the purpose of the study. All of them gave written informed consent to the study in accordance with the procedure approved by the ethics committee of Department of Neurological, Neurophysiological, Morphological, and Movement Sciences, University of Verona, Italy.

### Task

**Figure 1:**
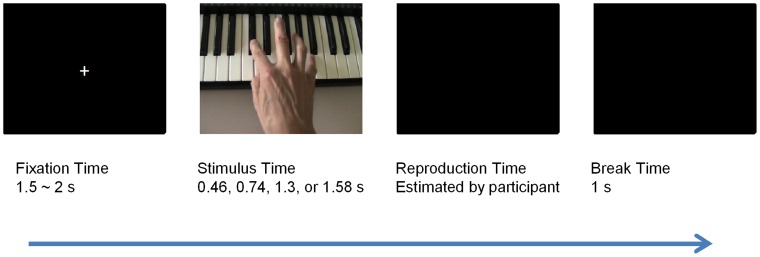
Procedure of a trial.

The task was a typical temporal duration reproduction set-up [23]. Participants were presented with the experimental stimulus, which was exposed for a certain temporal duration, and they had to reproduce as precisely as possible the same temporal duration by pressing the spacebar on the computer keyboard with their left big toe. The rationale of asking participants to use their toe to perform the task instead of their finger was to reduce the effect of the pianists’ expertise in finger movement execution. A fixation cross was displayed for a time that varied randomly within the range of 1500 and 1800 ms. The irregular fixation time ensured participants maintained their attention until the appearance of the next stimulus. At the end of the reproduction phase a black background was displayed for 1000 ms (see Figure 1).

### Materials and Procedure

Two video clips were created to represent a) a right hand performing piano-playing movements and b) the same hand performing finger-thumb opposition movements [17]. In both video clips the movements were taken from a bird’s eye view. For the piano-playing video clip, each finger pressed one key at a time on a typical piano keyboard, while for the finger-thumb opposition movements the hand was rotated 180 degrees, showing the palm of the hand to the observer, and the thumb pressed one finger tip at a time. In both video clips the finger movements were arranged in a randomized order with diverse finger movement combinations. The action was presented at a frequency of around 6 Hz. Moreover, a video-clip showing scrambled-pixels of the piano-playing video-clip was generated as a control stimulus. These three video clips were cut into four different lengths (460, 740, 1300 and 1580 ms). The time lengths were previously defined as to obtain two time windows below and two above the temporal duration of a second [21]. Additionally, from each aforementioned video-clip, we selected one frame and converted it into a “static image” (hereafter referred to as “picture”). The pictures were edited to last for the same four lengths of time as the video clips. Thus, there were 24 different experimental stimuli corresponding to the factorial combination of type of stimulus (piano playing, finger-thumb opposition, and scrambled-pixels), format (picture and video) and time length (460, 740, 1300, and 1580 ms). All of the stimuli were soundless and the action stimuli were presenting the same background: the piano keyboard. The experiment was conducted in a room insulated from external lights and noise. The stimulus was displayed on a black background and projected onto a transparent screen using a video beamer. Participants were seated about 1.2 m in front of the screen with the computer keyboard placed on the floor. The projected size of the stimulus was 1 m×1 m. They underwent a few practice trials to ensure that the task was fully understood and then they were prompted to initiate the actual experimental tests. No feedback was given.

Participants had to complete both action conditions (piano-playing and finger-thumb opposition) with the order of presentation counter-balanced within participants. In each condition, there were two different formats of stimuli presentation, each consisting of four different temporal lengths. The participants were later invited back in the laboratory and underwent a control condition in which the stimuli of scrambled-pixels were tested. Every temporal length was tested for 10 repetitions for a total of 240 trials. The experiment was divided into 12 sessions and within each session the testing order of the trials was randomised. A 5-minute break was given between sessions to avoid fatigue and task tediousness. The total experimental procedure took approximately three hours. The experimental program was written using MATLAB 7.1 and Cogent 2000.

### Data Analysis and Results

For each participant, data was first trimmed to discard outliers outside the range of the mean plus or minus twice the standard deviation. This procedure resulted in the loss of 3.1% of the entire set of data. The variables calculated were: (1) absolute error in a ratio with the respective stimulus lengths in order to evaluate participants’ reproduction error (from now on AE ratio), (2) the coefficient of variation of the reproduced times, calculated as the percentage of the standard deviation to the mean of the reproduction, to evaluate participants’ time reproduction variability (from now on CV). Statistical analyses were performed via Statistica 7.0. A significant main effect in the ANOVA was followed by Fisher LSD test as post hoc analysis. The significance level for all tests was set at *p*<0.050. For both variables of AE ratio and CV, a four-way repeated measures ANOVA (2 groups×3 types of stimulus×2 ranges of time×2 formats) with group (pianists and controls) as between-subjects factor and type of stimulus (piano playing, finger-thumb opposition and scrambled-pixels), range of time (sub-second and supra-second) and format (picture and video**-**clip) as within-subject factors. The results detected a significant main effect of group, suggesting that pianists represented a general tendency of being less erroneous, F [_1_], [_28_] = 17.439, *p*<0.001, and less variable, F [_1]_, [_28_] = 13.951, *p*<0.001, than the non-pianists. A significant main effect of range of time was also found, showing that when the time to be reproduced was shorter than one second, higher error, F [_1]_, [_28_] = 4.474, *p*<0.050, and higher variability, F [_1]_, [_28_] = 148.684, *p*<0.001, were caused. Moreover, we found a significant main effect of type of stimulus, F_ [2,56]_ = 4.682, *p*<0.050, for AE ratio and F_ [2,56]_ = 7.784, *p*<0.005, for CV. Participants showed less error and lower variability particularly for the scrambled-pixels stimuli than the other two actions stimuli. More importantly, there was a significant main effect of format, revealing that video-clip stimuli were provoking more estimation errors compared to the picture stimuli, F [_1]_, [_28_] = 5.457, *p*<0.050. Taken together, these results indicate that perceivers could process temporal duration using different mechanisms when viewing picture and video-clip visual displays. Therefore, two separated three-way repeated measures ANOVA (2 groups×3 types of action×2 ranges of time) were conducted for the picture and video-clip data, respectively.

### Picture Data

#### (a) Reproduction error (AE ratio)

**Figure 2:**
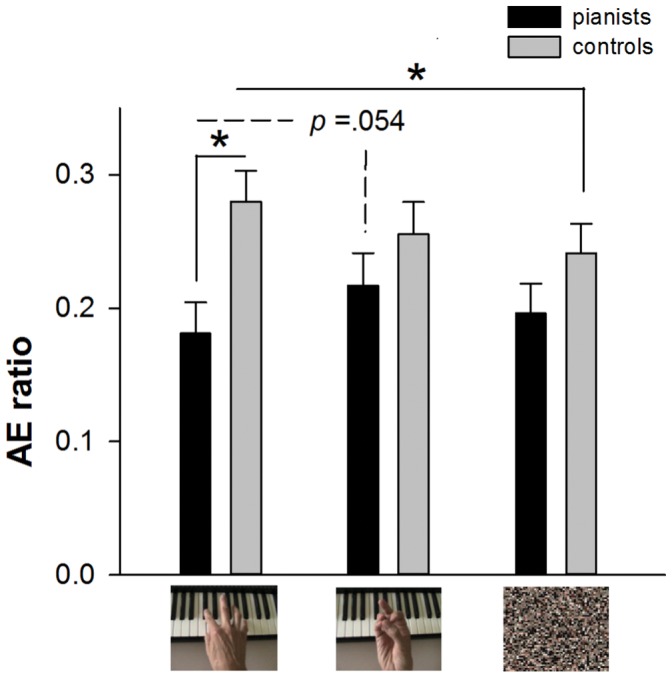
Average reproduction error (AE ratio) of pianists and controls for three types of pictures. Error bars indicate standard errors, * *p*<0.050.

The 3-way ANOVA (2 groups×3 types of stimulus×2 ranges of time) results detected a significant main effect of group, F [_1]_, [_28_] = 11.544, p<0.005, showing that the pianists produced less error compared to the controls (respective mean value of 0.198 and 0.259). Moreover, the main effect of group was found to significantly interact with the main effect of type of stimulus, F_ [2,56]_ = 3.284, p<0.050. Post-hoc analyses indicated that the pianists were less erroneous while they were viewing the piano-playing actions than when viewing the finger-thumb opposition movements, p value approached to significance level (p = 0.054). On the other hand, the control group did not show a difference in their reproduction error for the two types of actions, p = 0.188, and they produced greater error for piano-playing than for scrambled-pixels pictures. Moreover, specifically for the piano-playing actions, the pianists outperformed the controls, p<0.010, whereas no difference was found between groups for the finger-thumb opposition action, p = 0.225 and for the scrambled-pixels pictures, p = 0.157 (See Figure 2). The main effect of type of stimulus, F_ [2,56]_ = 0.973, p = 0.384, and range of time, F [_1]_, [_28_] = 0.333, p = 0.568, and the other interactions were not significant, all p values >0.050.

#### (b) Reproduction variability (CV)

**Figure 3:**
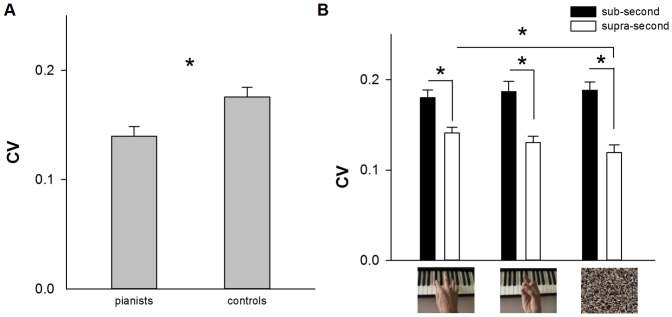
Average reproduction variability (CV) for picture stimuli. Panel A shows the difference between pianists and controls; panel B shows the difference when the exposure time of pictures was shorter and longer than a second. Error bars indicate standard errors, * *p*<0.050.

The results of the 3-way ANOVA (2 groups×3 types of stimulus×2 ranges of time) yielded a significant main effect of group, F [_1]_, [_28_] = 8.110, p<0.010, in that pianists performed the task globally with less variability than the control group (respectively mean values of 0.140 and 0.176; see Figure 3 panel A). The main effect of range of time was also found significant, F [_1]_, [_28_] = 96.241, p<0.001, with sub-second stimuli reproduced with higher variability than supra-second ones (respective mean values of 0.185 and 0.130). Moreover, the main effect of range of time significantly interacted with the main effect of type of stimulus, F_ [2, 56]_ = 4.013, p<0.050. Post-hoc comparisons indicated that when the time to be reproduced was shorter than one second, participants did not differentiate the three types of stimulus. Whereas when the time to be reproduced was longer than one second, piano-playing pictures were reproduced with higher variability than the scrambled-pixels ones, p<0.010 (See Figure 3 panel B). The main effect of type of stimulus was not detected significant, F_ [2,56]_ = 0.413, p = 0.663. The other interactions were not significant, all **p values >0.050**.

### Video Data

#### (a) Reproduction error (AE ratio)

**Figure 4:**
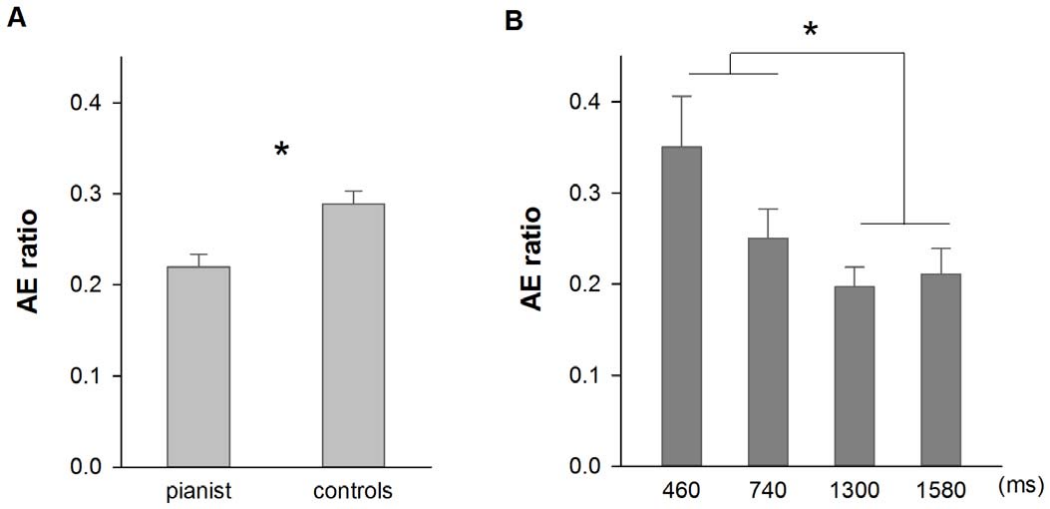
Average reproduction error (AE ratio) for video-clip stimuli. Panel A shows the difference between pianists and controls; panel B shows the difference when the exposure time of video-clips was shorter (460 and 740 ms) and longer than a second (1300 and 1580 ms). Error bars indicate standard errors, * *p*<0.050.

Similarly, from the results of the 3-way ANOVA (2 groups×3 types of stimulus×2 ranges of time) we found a significant difference between pianists and controls, F [_1]_, [_28_] = 11.686, p<0.010 (mean value of 0.220 and 0.289 for pianists and controls respectively; see Figure 4 panel A). The main effect of range of time was found significant, F [_1]_, [_28_] = 8.522, p<0.050, with sub-second time durations (460 and 740 ms) reproduced with greater error than the supra-second ones (1300 and 1580 ms); respective mean value of 0.303 and 0.206 (See Figure 4 panel B). The main effect of type of stimulus was also found significant, F_ [2,56]_ = 5.424, p<0.010, with finger-thumb opposition video-clips were reproduced with greater error compared to the scrambled-pixels ones (respective mean of 0.287 and 0.221; 0.256 for the piano-playing video-clips). All of the interactions were not significant, all p values >0.050.

#### (b) Reproduction variability (CV)

**Figure 5:**
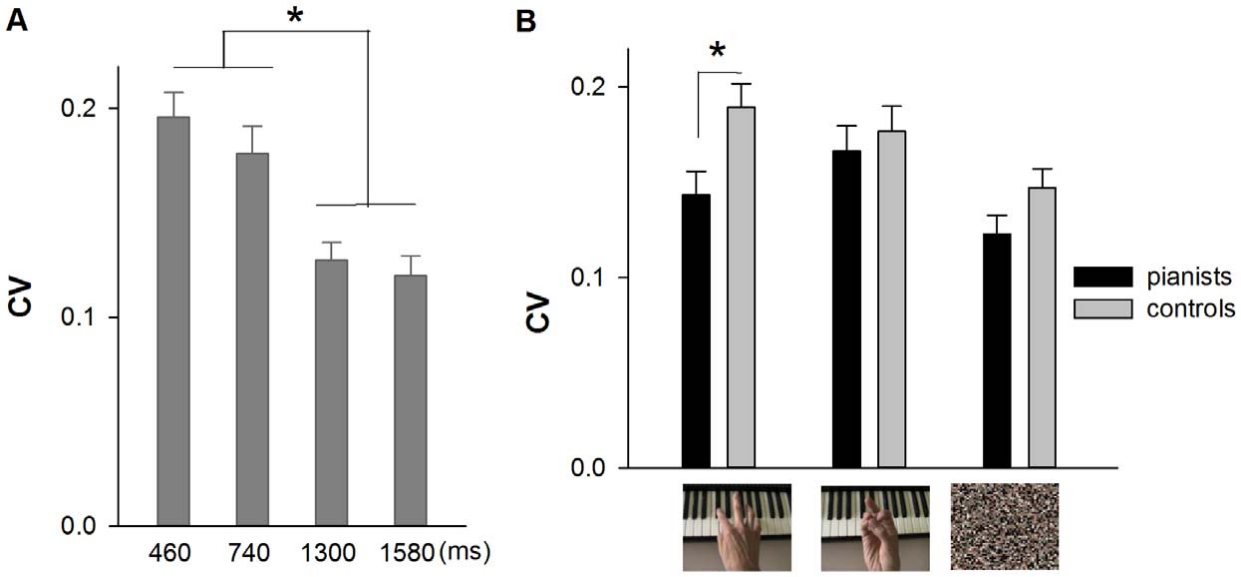
Average reproduction variability (CV) for video-clip stimuli. Panel A shows the difference when the exposure time of video-clips was shorter (460 and 740 ms) and longer than a second (1300 and 1580 ms); panel B shows the difference between pianists and controls while they viewed the three different types of video-clips. Error bars indicate standard errors, * *p*<0.050.

The 3-way ANOVA (2 groups×3 types of stimulus×2 ranges of time) detected a significant main effect of group, F [_1]_, [_28_] = 14.953, p<0.001, indicating that the pianists performed the task with lower variability compared to the controls (respective mean value 0.144 and 0.171). We also found a significant main effect of range of time, showing that sub-second durations (460 and 740 ms) were reproduced with higher variability than the supra-second ones (1300 and 1580 ms), F [_1]_, [_28_] = 88.995, p<0.001 (See Figure 5 panel A). The main effect of type of stimulus was also significant, F_ [2, 56]_ = 10.883, p<0.001, with scrambled-pixels stimuli were reproduced with the least variability among the three ones, p values <0.001. The group-by-type of stimulus interaction was not found significant, F_ [2 56]_ = 2.231, p = 0.117. However, while looking into the post-hoc comparisons we found that pianists presented lower reproduction variability than the controls only when they viewed piano-playing video-clips, p<0.001 (See Figure 5 panel B). Moreover, they tended to differentiate the piano-playing video-clips with the finger-thumb opposition ones, p value approached to the significance level (p = 0.061). The controls reproduced the lowest variability for the scrambled-pixels video-clips than for the other two action video-clips, p values <0.005; while the two action video-clips were not differentiated, p = 0.297. The other interactions were not significant, p values far from significance level.

## Discussion

The aim of this study was to investigate whether observing a highly familiar action triggers more precise internal clocks for temporal duration estimation. We thus investigated pianists’ estimates in reproducing different times of exposure of piano-playing and non-piano-playing (i.e., finger-thumb opposition and scrambled-pixels) visual displays presented either in photograph or in video-clip format and compared their performance with non-pianists.

Both error and variability have been used to evaluate participants’ estimates of temporal duration, the former measuring the capacity to remain as close as possible to the target duration (accuracy), and the latter computing the dispersion of estimates around the target over trials (stability) [24]–[25]. Thus less error and lower variability indicate higher estimation proficiency. Indeed, we found overall that pianists were better than non-pianists in terms of both accuracy and stability. Remarkably, the group difference was particularly magnified by the pianists’ enhanced performance only for pictures and video-clips showing the hand in the act of playing the piano; when instead the action observed was a finger-thumb opposition or when it was a scrambled-pixels display, pianists and non-pianists showed the same performance. Overall these results suggest that a highly detailed motor experience and familiarity about an action triggers highly precise internal clocks even though probably both attention-related cues along with action-specific internal simulations are involved. Therefore, besides the very well known notion that an internal action simulation leads to a better understanding and preplanning of that action [7]–[16], [17]–[20], [26]–[27], our findings add the idea that an internal action simulation enhances the sense of temporal duration as well [4]. In the same vein, non-pianists, who had not developed a specific internal action simulation either in terms of visual familiarity or in terms of motor ability, showed no difference in temporal duration evaluation with the piano-playing or the finger-thumb opposition actions.

This study for the first time investigated temporal duration evaluation with actions represented either in a picture or in a sequence of pictures forming a video-clip. Since the two visual conditions might involve different internal timing mechanisms, we hypothesized that when the action is “implied” [11]–[15], as in a static image, the timing or “pace” of the internal embodiment can be defined by the individual observer. On the contrary, the observer’s internal timing might be influenced by the observation of a given action sequence such as the one represented in a video-clip, where the pace is “apparent” to the observer [16]–[19]. Indeed we found that individuals were more accurate in evaluating temporal duration when they were exposed to static images compared to sequences of images (video-clips). In particular the video-clips, which were presented with an exposure time of less than one second, produced the highest error for both groups. This result is sustained by the general observation that in order to develop a specific internal action simulation, a movement sequence needs to be observed for at least 800 ms or more [7], [17]–[19]. The short time provided in our study was probably not long enough to form an internal action simulation, thus not allowing observers to present the same level of accuracy as they revealed while viewing the photos. It could be that when the time of exposure is too short (especially for the durations below a second) the temporal and spatial changes conveyed by the video-clip are perceived as too fast and as a consequence they can act as noise, which distorts individuals’ perception of temporal duration. Indeed, previous time estimation studies have also reported an enlarged bias of overestimation in evaluating short durations due to the greater spatial changes presented in the visual stimuli [23]–[28]. Nevertheless, pianists demonstrated a lower variability in their performance for piano-playing video-clips than non-pianists. This may be because pianists were able to counteract the perturbations given by the short exposure to the movement by stabilizing their estimates, particularly when viewing piano-playing actions that they have been intensively trained to perform. To sum up, pianists appeared to apply two different strategies: for picture stimuli they were able to estimate the temporal duration more precisely, while for video-clip stimuli they were able to estimate the time more consistently and these two strategies were enhanced particularly when evaluating piano-playing actions. Here we furthermore determine that this time distortion, usually tested and obtained by using simple and meaningless geometric patterns, also holds for the observation of complex and meaningful patterns such as human actions.

It is also important to notice that overall, all the participants tested - both pianists and non-pianists - presented more error and a higher variability when estimating sub-second durations compared to supra-second ones. This result was in accordance with previous reports in time reproduction in showing that variability monotonically decreases as the target time to be estimated increases [29]–[31]. This suggests a higher demand of perceptual-motor capabilities when estimating brief temporal duration. However, pianists demonstrated less estimation error and lower variability than non-pianists even in sub-second exposure times. Their superior clock in such a brief time window can be attributed to a more efficient “automatic” sensory motor system developed by extensive musical training [32]–[33]. Pianists also showed better temporal duration evaluation compared to non-pianists when estimating times of over one second. It could well be more cognitive allowance such as working memory capacity developed through musical training [34], which helped them to concentrate better and adopt music-related strategies. Therefore, our results suggest that the highly precise functioning of an internal action simulation such as that possessed by pianists is robust no matter how long they viewed the stimuli: above or below a second. Notably, by asking participants to press the computer key with their left foot, we avoided the bias that could be present due to pianists’ ability and familiarity with finger movements.

Taken together, our results indicate that forming an internal action simulation is relevant not only for a social understanding of other people’s actions [16]–[20], for skill acquisition [20] or for action imitation [35], but also more subtly for timing specific movements [4]. We show that this internal mechanism is particularly enhanced in individuals with a highly developed motor sensory system. In other words, the more the observed action is familiar and well known at the level of performance, the easier it is to estimate its temporal duration. This inference is consistent with previous findings that motor experience improves precision in action anticipation [7]–[8] as, for example, with string musicians in predicting the entry of sound produced by a violin when compared to non-string musicians and non-musicians [9]. Furthermore, the active and dynamical internal action simulation seems to operate more efficiently when observers are allowed to form and pace the actions freely such as when viewing an implied action. When instead the action is represented by a sequence of images as in a video-clip, the time of exposition is critical in whether it allows an internal action simulation to develop or not. When the exposure time is too short (for example, below a second) temporal duration evaluation decreases in accuracy due to the fast changes of the movements presented in the space-time domain. Nevertheless it was remarkable to see that even in such difficult situations pianists were able to counteract these perturbations by maintaining a higher stability across trials and in particular for the action pertaining to their domain of expertise.

In conclusion, our results for the first time provide evidence for the idea that through musical training, pianists create a selective and self-determined internal action simulation of an observed movement that allows them to estimate precisely its temporal duration.
